# The effect of parathyroidectomy on bone mineral density in primary hyperparathyroidism

**DOI:** 10.3906/sag-1904-49

**Published:** 2019-12-16

**Authors:** Mustafa ÇALIŞKAN, Selvihan BEYSEL, Muhammed KIZILGÜL, Mustafa ÖZBEK, Erman ÇAKAL

**Affiliations:** 1 Department of Endocrinology and Metabolism, Atatürk Düzce State Hospital, Düzce Turkey; 2 Department of Endocrinology and Metabolism, Afyonkarahisar Sağlık Bilimleri University, Afyonkarahisar Turkey; 3 Department of Endocrinology and Metabolism, University of Health Sciences,Dışkapı Yıldırım Beyazıt Teaching and Research Hospital, Ankara Turkey

**Keywords:** Bone mineral density, parathyroidectomy, hyperparathyroidism

## Abstract

**Background/aim:**

This study aimed to investigate the change in bone mineral density (BMD) before and 1 year after parathyroidectomy in patients with primary hyperparathyroidism (PHPT).

**Materials and methods:**

The clinical and biochemical parameters and DEXA screening of patients with symptomatic PHPT (n = 28) and asymptomatic PHPT (n = 63) were investigated before and 1 year after parathyroidectomy.

**Results:**

Patients with symptomatic PHPT had a higher prevalence of nephrolithiasis (18.2% vs. 4.6%, P = 0.032) when compared to the prevalence in patients with asymptomatic PHPT. The prevalence of osteoporosis in the lumbar spine (63.0% vs. 37.5%, P = 0.026) and femoral neck (40.7% vs. 20.6%, P = 0.048) was higher in symptomatic PHPT when compared to the prevalence in asymptomatic PHPT. After parathyroidectomy, the decreases in the prevalence of osteoporosis in the lumbar spine (25.8% vs. 9.4%, P = 0.014), femoral neck (22.1% vs. 8.2%, P =0.009), and total hip (22.4% vs. 5.3%, P = 0.007) were higher in symptomatic PHPT compared to the asymptomatic PHPT group. A higher BMD gain (g/cm^2^) was seen in the lumbar spine (10.83% vs. 4.65%, P=0.016) and femoral neck (12.61% vs. 4.37%, P=0.005) in symptomatic PHPT compared to the asymptomatic PHPT group.

**Conclusion:**

Parathyroidectomy provided more BMD gain in the lumbar spine and femoral neck in patients with symptomatic PHPT when compared to patients with asymptomatic PHPT 1 year after parathyroidectomy.

## 1. Introduction

Patients with elevated serum parathyroid hormone (PTH) and calcium levels are defined as having primary hyperparathyroidism (PHPT). Asymptomatic primary PHPT is characterized as a disorder without signs or symptoms associated with PTH and calcium excess [1]. Many patients with asymptomatic PHPT report nonspecific symptoms such as weakness, depression, anxiety, fatigue, loss of initiative, disturbed sleep, decreased memory and cognitive function, constipation, polydipsia, and pain in the extremities [1,2]. Nowadays, classic features of PHPT such as stones, abdominal groans, psychic moans, and fragile fractures of bones are not commonly observed because of routine biochemical analyses [2]. Most cases of PHPT are discovered incidentally upon biochemical testing when patients are mild and asymptomatic [3]. The disease presents more subtly; therefore, surgery management remains a clinical dilemma. The National Institutes for Health (NIH) consensus guidelines for the management of asymptomatic PHPT recommend surgery in the event of serum calcium of ≥1 g/dL above the upper limit of normal or a previous fragility fracture or an energy X-ray absorptiometry (DEXA) score of less than –2.5 at any site or estimated glomerular filtration rate of <60 mL/min or the patient is aged <50 years or medical follow-up is undesired or impractical [2].

PHPT is associated with osteoporosis/osteopenia; however, overt complications of the skeleton are rarely encountered nowadays [1]. Overt complications of the disease such as osteitis fibrosa cystica, salt-and-pepper sign, periosteal bone resorption, bone cysts, and brown tumors are commonly seen in classic PHPT [2]. These findings generally are not observed in asymptomatic PHPT but DEXA and bone biopsies reveal skeletal involvement. Both trabecular and cortical bones are affected in asymptomatic patients with PHPT [3]. Epidemiologic studies have revealed an increased risk of both vertebral and nonvertebral fractures in patients with PHPT [4,5]. Increased bone turnover [6], decreased bone mineral density (BMD) score [7], and increased risk of fragile fracture [8] have been observed in asymptomatic patients PHPT, even in the early phase of the disease. BMD measurement in the lumbar spine, a trabecular site, is relatively preserved, whereas the distal one-third of the radius, a cortical site, is reduced. PHPT has a greater catabolic effect on the cortical bone but a relatively sparing effect on trabecular bone. Bone remodeling slows down, and the BMD score subsequently increases and fragile bone fractures decrease after a biochemical cure provided by a successful parathyroidectomy [6,9–13]. The BMD scores are improved after successful parathyroidectomy in patients with mild PHPT [12,14]. Successful parathyroidectomy in symptomatic PHPT increases BMD the most, and the fastest is in the lumbar spine and hip, followed later by increases in the distal 1/3 of the radius. PHPT patients with severe skeletal involvement benefit more from successful parathyroidectomy [15–18]. However, the effect of surgical treatment on the skeleton in asymptomatic PHPT remains unclear. This study aimed to investigate the change in BMD before and 1 year after parathyroidectomy in patients with PHPT. 

## 2. Materials and methods

Patients with PHPT who were referred to our tertiary hospital (Ankara, Turkey), in the Department of Endocrinology and Metabolism, from 2011 to 2014, were recruited for this prospective study. Most of the patients presented with osteoporosis and symptoms unrelated to hyperparathyroidism. Each patient gave informed consent as per the Declaration of Helsinki. This study was approved by the Dışkapı Yıldırım Beyazıt Teaching and Research Hospital Ethics Board (Number: 24.02.2013-12/21).

We tested for symptoms related to hypercalcemia (repeated nephrolithiasis, gastritis, polyuria, muscle weakness, osteoporosis, or psychiatric disorders) and bone fractures. Patients with a hypercalcemic crisis, defined as serum calcium of ≥14 mg/dL, were not included. Patients with Cushing’s syndrome, osteomalacia, renal insufficiency, hepatic disorders, rheumatoid arthritis, and ankylosing spondylitis were excluded. No patients used bisphosphonates, calcimimetic agents, hormone replacement treatment, selective estrogen receptor modulators, or cortisol before the start of the study or after parathyroidectomy. However, five patients had hungry bone syndrome following parathyroidectomy, and five patients started taking active vitamin D3 (calcitriol) soon after parathyroidectomy. Three patients began treatment with calcium acetate, and two patients used both. Patients who had serum vitamin D of <20 ng/mL were given vitamin D3 at 50,000 IU/week for 8 weeks and subsequent daily oral dosages of 800 IU vitamin D3 after parathyroidectomy. Patients who had fragile fractures were not included. 

We excluded patients without follow-up (n = 19) from the study. In the end, we only included 91 patients with complete data in the study. We stratified patients into two groups as having symptomatic PHPT (n = 28) or asymptomatic PHPT (n = 63). Asymptomatic primary hyperparathyroidism described patients who lacked obvious signs and symptoms associated with either elevated calcium or parathyroid hormone. We included patients with asymptomatic PHPT requiring parathyroidectomy in this study. We set the criteria for parathyroidectomy as in the NIH recommendations [2]. A single surgical group performed all operations. All patients had the following characteristics: a) biochemical and clinical diagnosis of PHPT, b) successful parathyroidectomy as confirmed by normal postparathyroidectomy serum calcium levels, and c) follow-up with clinical, biochemical, and BMD assessment before and after parathyroidectomy. 

Clinical, biochemical, and anthropometric measurements were performed before and after parathyroidectomy. Body mass index (BMI) was calculated as weight (kg) / height (m2). Serum calcium, phosphorus, 25-hydroxy vitamin D3 (25OHD), intact parathyroid hormone (iPTH), alkaline phosphatase (ALP), thyroid-stimulating hormone (TSH), creatinine, albumin, glucose, and urinary calcium excretion were measured. We measured serum iPTH using a radioimmunoassay (DiaSorin Inc., Stillwater, MN, USA). We checked successful parathyroidectomies in all patients at 3, 6, and 12 months through normalization of serum calcium. We used DEXA and renal ultrasound to determine the advisability of surgery in asymptomatic patients and to monitor patients. Clinically silent nephrolithiasis and nephrocalcinosis were detected using renal ultrasonography before parathyroidectomy while investigating the criteria for surgery. BMD measurements in the lumbar spine (L1–L4), hips, and femoral neck were performed before and after parathyroidectomy using DEXA (Hologic QDR-4500 device, Hologic Inc., Waltham, MA, USA). BMD is expressed in grams per square centimeter (g/cm^2^) and as standard deviations (T-scores). BMD change (%) was assessed using the following formula: (postoperative – preoperative BMD / preoperative BMD) × 100 [17]. Osteoporosis was diagnosed as a BMD T-score of less than or equal to –2.5 SD in the lumbar spine, hip, or femoral neck [2]. 

### 2.1. Statistical analysis

Statistical analysis was performed using SPSS 18.0 (SPSS Inc., Chicago, IL, USA). Variables are presented as mean ± standard deviation (SD) or median (min–max), percentage (%), odds ratio (OR), and 95% confidence interval (CI). Normality was tested using Kolmogorov–Smirnov and Shapiro–Wilk W tests. The chi-square test or Fisher’s exact test was used for categorical variables. Student’s t-test was used for normally distributed continuous variables. The Mann–Whitney U test was used for continuous variables that were not normally distributed. McNemar’s test was used for categorical variables between preparathyroidectomy and postparathyroidectomy values. The paired-samples t-test was used for normally distributed continuous variables between preparathyroidectomy and postparathyroidectomy. The Wilcoxon test was used for continuous variables that were not normally distributed between preparathyroidectomy and postparathyroidectomy. Logistic regression analysis was performed: BMD gain was defined as the dependent variable and preparathyroidectomy variables such as adenoma volume, iPTH, ALP, and symptoms were independent variables. Variables identified with a P-value of ≤0.1 were entered in the model. Statistical significance was defined as P < 0.05. 

## 3. Results

Among the patients with PHPT, 69.2% were asymptomatic. Sex, menopause, age, BMI, and smoking habits were similar between groups (P > 0.05). Patients with symptomatic PHPT had a higher prevalence of nephrolithiasis (18.2% vs. 4.6%, P = 0.032) and higher adenoma volume (2.95 ± 3.49 vs. 0.47 ± 0.56 cm3, P = 0.027) than seen in the asymptomatic PHPT group. Serum calcium, ALP, and iPTH were higher in patients with symptomatic PHPT than in patients with asymptomatic PHPT (P < 0.05). Levels of 25(OH)D and phosphorus were lower in patients with symptomatic PHPT than in patients with asymptomatic PHPT (P < 0.05). The number of patients with at least one site of osteoporosis was higher in symptomatic PHPT patients than in patients with asymptomatic PHPT (66.7% vs. 39.1%, P = 0.016). The number of patients with osteoporosis in the lumbar spine (63.0% vs. 37.5%, P = 0.026) and femoral neck (40.7% vs. 20.6%, P = 0.048) was higher for symptomatic PHPT than asymptomatic PHPT. Osteoporosis in the total hip did not differ between the groups (P > 0.05). Total hip and BMD-T score in the lumbar spine was higher in patients with symptomatic PHPT than in patients with asymptomatic PHPT (–2.92 ± 1.17 vs. –1.99 ± 1.41, P = 0.020). The total hip and femoral neck BMD T-sore did not differ between the groups (P > 0.05). We detail the clinical parameters of patients with asymptomatic PHPT or symptomatic PHPT before parathyroidectomy in Table 1. 

**Table 1:** Clinical, biochemical, and bone mineral densitometry parameters of patients with asymptomatic and symptomatic primary hyperparathyroidism before parathyroidectomy.

Variables	Asymptomatic PHPT, n = 63	Symptomatic PHPT, n = 28	P
Women (%)	82.8	77.8	0.314
Menopause (%)	61.5	61.9	0.815
Smoking habit (%)	20.6	25.9	0.715
Age (years)	51.52 ± 10.47	52.40 ± 11.59	0.826
BMI (kg/m^2^)	29.95 ± 4.72	32.13 ± 6.60	0.482
Glucose (mg/dL)	90.85 ± 9.99	89.60 ± 9.01	0.934
Albumin (mg/dL)	4.53 ± 0.22	4.41 ± 0.15	0.352
Creatinine (mg/dL)	0.75 ± 0.15	0.68 ± 0.16	0.408
Calcium (mg/dL)	10.41 ± 0.64	11.34 ± 0.76	<0.001
Phosphorus (mg/dL)	2.61 ± 0.37	2.50 ± 0.44	0.030
iPTH (pg/dL)	194.76 ± 136.63	347.53 ± 202.24	<0.001
ALP (mg/dL)	101.52 ± 40.08	158.20 ± 74.90	<0.001
25(OH)D (ng/mL)	20.15 ± 24.11	9.82 ± 9.13	0.003
Magnesium (mg/dL)	2.13 ± 0.19	2.12 ± 0.23	0.935
TSH (mg/dL)	2.09 ± 1.29	1.56 ± 0.81	0.728
Lumbar spine BMD (g/cm^2^)	0.83 ± 0.15	0.72 ± 0.12	0.137
Total hip BMD (g/cm^2^)	0.78 ± 0.13	0.71 ± 0.15	0.951
Femoral neck BMD (g/cm^2^)	0.67 ± 0.11	0.63 ± 0.09	0.879
Lumbar spine T-score	–1.99 ± 1.41	–2.92 ± 1.17	0.020
Total hip T-score	–1.44 ± 1.14	–1.98 ± 1.30	0.242
Femoral neck T-score	–1.83 ± 1.17	–2.32 ± 1.09	0.165
Osteoporosis at lumbar spine (%)	37.5	63.0	0.026
Osteoporosis at total hip (%)	12.5	29.6	0.050
Osteoporosis at femoral neck (%)	20.6	40.7	0.048
Osteoporosis at least one site (%)	39.1	66.7	0.016
Nephrolithiasis (%)	4.6	18.2	0.032
Urinary calcium excretion (mg/24 h)	362.61 ± 175.20	437.33 ± 87.18	0.016
Adenoma volume (cm^3^)	0.47 ± 0.56	2.95 ± 3.49	0.027

Continuous variables are presented as mean ± standard deviation (SD). Categorical variables are presented as percentage (%). BMI, Body mass index; TSH, thyroid-stimulating hormone; ALP, alkaline phosphatase; iPTH, intact parathyroid hormone, BMD, bone mineral density; 25(OH)D, 25-hydroxy-vitamin D.

### 3.1. Bone mineral density gain at one year of parathyroidectomy

After successful parathyroidectomy, serum calcium, iPTH, and ALP were more decreased in patients with symptomatic PHPT than in patients with asymptomatic PHPT (P < 0.05). Magnesium, 25(OH)D, phosphorus, and BMI did not change between the groups (P > 0.05). The decrease in the number of patients with at least one site of osteoporosis was higher in patients with asymptomatic PHPT than in patients with symptomatic PHPT (22.1% vs. 6.3%, P = 0.008). The decrease in the prevalence of osteoporosis in the lumbar spine (25.8% vs. 9.4%, P = 0.014), femoral neck (22.1% vs. 8.2%, P = 0.009), and total hip (22.4% vs. 5.3%, P = 0.007) was higher in patients with symptomatic PHPT than in patients with asymptomatic PHPT. A higher BMD gain (g/cm^2^) was seen in the lumbar spine (10.83% vs. 4.65%, P = 0.016) and femoral neck (12.61% vs. 4.37%, P = 0.005) in patients with symptomatic PHPT when compared to patients with asymptomatic PHPT. Total hip BMD change (g/cm^2^) did not differ between the groups (6.57% vs. 3.75%, P > 0.05). The increase in BMD T-score was higher in the lumbar spine (0.65 ± 0.60 vs. 0.30 ± 0.77, P = 0.039), femoral neck (0.98 ± 0.94 vs. 0.36 ± 0.67, P = 0.001), and total hip (0.54 ± 0.55 vs. 0.26 ± 0.51, P = 0.021) in patients with symptomatic PHPT than in patients with asymptomatic PHPT. Change in clinical parameters after parathyroidectomy is shown in Table 2.

**Table 2:** Change in clinical parameters after parathyroidectomy.

Variables	Asymptomatic PHPT, n = 63	Symptomatic PHPT, n = 28	P
∆ BMI (kg/m^2^)	0.48 ± 1.31	0.19 ± 0.85	0.282
∆ Calcium (mg/dL)	–1.43 ± 0.68	-3.32 ± 5.82	0.012
∆ Phosphorus (mg/dL)	0.73 ± 0.49	-0.89 ± 0.52	0.168
∆ iPTH (pg/dL)	–128.71 ± 122.86	-297.51 ± 310.81	<0.001
∆ ALP (mg/dL)	–22.82 ± 27.06	-78.25 ± 66.08	<0.001
∆ Magnesium (mg/dL)	–0.13 ± 0.27	0.03 ± 0.24	0.486
∆ 25(OH)D	14.84 ± 24.12	19.01 ± 9.17	0.379
∆ Lumbar spine BMD (g/cm^3^) %	4.65 (–10.00 to 30.65)	10.83 (-6.72 - 70.08)	0.016
∆ Total hip BMD (g/cm^3^) %	3.75 (–11.57 to 21.29)	6.57 (-7.53 - 51.98)	0.124
∆ Femoral neck BMD (g/cm^3^) %	4.37 (–11.21 to 30.04)	12.61 (-14.17 - 54.85)	0.005
∆ Lumbar spine BMD T-score	0.30 ± 0.77	0.65 ± 0.60	0.039
∆ Total hip BMD T-score	0.26 ± 0.51	0.54 ± 0.55	0.021
∆ Femoral neck BMD T-score	0.36 ± 0.67	0.98 ± 0.94	0.001
∆ Osteoporosis at lumbar spine (%)	25.8	9.4	0.014
∆ Osteoporosis at total hip (%)	22.4	5.3	0.007
∆ Osteoporosis at femoral neck (%)	22.1	8.2	0.009
∆ Osteoporosis of at least one site (%)	22.1	6.3	0.008

Continuous variables are presented as mean ± standard deviation (SD). Categorical variables are presented as percentage (%). Difference (Δ) is shown as change in values before and after treatment (latter value minus former value). BMI, Body mass index; ALP, alkaline phosphatase; iPTH, intact parathyroid hormone, BMD, bone mineral density; 25(OH)D, 25-hydroxy-vitamin D.

Univariate analysis showed that BMD gain in the lumbar spine and femoral neck was positively correlated with preparathyroidectomy iPTH, pre-ALP, adenoma volume, and being in the symptomatic PHPT group (Table 3). In the logistic regression analysis, preparathyroidectomy iPTH was the only variable identified as an independent predictor of BMD gain (β = 3.18, 95% CI: 2.3–13.5, P = 0.02). 

**Table 3:** Correlation between preparathyroidectomy variables and BMD gain at lumbar spine and femoral neck

	BMD gain in lumbar spine		BMD gain in femoral neck	
	r*	P	r*	P
Pre-iPTH Pre-ALP Adenoma volume Symptomatic PHPT group	0.338 0.319 0.396 0.276	0.124 0.148 0.104 0.015	0.329 0.580 0.201 0.321	0.135 0.005 0.423 0.004

r* represents correlation coefficient. ALP, Alkaline phosphatase; iPTH, intact parathyroid hormone.

## 4. Discussion 

Our study showed that osteoporosis was more prevalent in patients with symptomatic PHPT than in patients with asymptomatic PHPT. Parathyroidectomy reduced the prevalence of osteoporosis in the lumbar spine, femoral neck, and total hip with biochemical cure in both patients with symptomatic PHPT and asymptomatic PHPT 1 year after parathyroidectomy. Parathyroidectomy provided a better BMD gain in the lumbar spine and femoral neck in patients with symptomatic PHPT compared to patients with asymptomatic PHPT. Preparathyroidectomy iPTH was an independent predictor of BMD gain. 

Trabecular and cortical bone mineral density were decreased in patients with asymptomatic PHPT and symptomatic PHPT [3,19–22]. Our study showed a higher prevalence of osteoporosis in the lumbar spine and femoral neck in patients with symptomatic PHPT than in patients with asymptomatic PHPT; however, total hip osteoporosis was similar between the groups. After a successful parathyroidectomy, BMD scores were increased in symptomatic PHPT cases [6,9–11]. Parathyroidectomy has improved BMD scores in patients with mild PHPT [12,14]. Successful parathyroidectomy increased BMD scores the most and fastest in the lumbar spine and hip followed later by increases in radius, especially in symptomatic PHPT. Parathyroidectomy has improved severe skeletal involvement in symptomatic PHPT [15–18]. Our results show that BMD loss in the femoral neck and lumbar spine was higher in patients with symptomatic PHPT than in patients with asymptomatic PHPT. After parathyroidectomy, BMD gain in the femoral neck and lumbar spine was higher in patients with symptomatic PHPT than in patients with asymptomatic PHPT. In our study, we observed that surgery may improve bone involvement even in asymptomatic PHPT with osteopenia; therefore, we suggest that parathyroidectomy may be useful for the treatment of osteopenia/osteoporosis in asymptomatic PHPT patients. 

A study showed that BMD score decreased in the femoral neck and remained unchanged in the lumbar spine in an observation group, whereas BMD improved in the lumbar spine and femoral neck in the parathyroidectomy group [23]. They observed bone loss in the hips in asymptomatic PHPT cases without surgery [24]. Parathyroidectomy was thought to be more cost-effective than observation without treatment in patients with mild asymptomatic PHPT [25]. Postoperative BMD gain in the femoral neck and total hip with decreasing bone-specific alkaline phosphatase concentrations have been reported in patients with mild asymptomatic PHPT [12,26,27]. Our results showed that BMD increased by 10.83% in the lumbar spine, by 6.57% in the total hip, and by 12.61% in the femoral neck in patients with symptomatic PHPT after parathyroidectomy while BMD increased by 4.65% in the lumbar spine, 3.75% in the total hip, and 4.37% in the femoral neck in patients with asymptomatic PHPT after parathyroidectomy. However, BMD gain in the total hip was similar in the two groups. Several studies have reported significant BMD gain following parathyroidectomy [14–17,26,28]. The BMD was lower in patients with severe PHPT and significant improvements were observed after surgical cure, which is consistent with our results [17,29]. Skeletal abnormalities regressed and osteoclastoma shrunk with BMD gain following successful parathyroidectomy in severe PHPT [9,30,31]. Significant and rapid BMD gain was observed in patients with osteitis fibrosa cystica, whereas slow BMD gain was observed in patients with asymptomatic PHPT [11,30,31]. During follow-up over 2 years, stable BMD gain was observed in PHPT patients who underwent parathyroidectomy, whereas slow bone loss was observed in those without parathyroidectomy [32]. BMD gain after surgery in asymptomatic PHPT was reported during 1–3 years of follow-up [12,14,26]. After a successful parathyroidectomy, significant and rapid BMD gain was observed in the lumbar spine and hip, followed by BMD gain in the radius [11,27,28]. Long-lasting stable BMD gain was reported at all bone sites after successful parathyroidectomy in asymptomatic PHPT [10,11,28]. After successful parathyroidectomy, BMD gain at 5, 10, and 15 years was observed; however bone loss in the hip and radius was observed in patients without parathyroidectomy [11]. Our results showed that BMD was improved in both patients with symptomatic PHPT and asymptomatic PHPT after successful parathyroidectomy.

Postoperative BMD gain shows variations among patients with PHPT. Changes in serum PTH concentration, initial bone turnover, age, sex, and preoperative vitamin D status are considered effective for postoperative bone gain [12,18]. Our study showed that symptomatic PHPT cases had lower serum 25(OH)D level than asymptomatic PHPT. Changes in 25(OH)D and BMI did not differ between the groups following parathyroidectomy. Our study showed that BMD gain in the lumbar spine and femoral neck was positively correlated with pre-iPTH, pre-ALP, adenoma volume, and having symptomatic PHPT. Preparathyroidectomy iPTH was the only independent predictor of BMD gain. These findings may suggest that BMD gain might be more remarkable in patients with higher preoperative iPTH. Similarly, a study showed that postoperative BMD gain was correlated with preoperative iPTH [18]. Decreased PTH concentrations lead to decreased activation of new bone remodeling such as bone turnover and remodeling space [15,18]. Chronically high PTH enhances osteoclastic bone resorption and bone turnover. PTH plays a critical role in PTH receptors on osteoblasts, which stimulate the osteoclastic differentiation of osteoblasts and lead to cortical bone resorption [33]. Postoperative BMD gain could be explained by decreasing serum iPTH concentration.

The present study has a small sample size and short prospective follow-up, a control group was not used for comparison, and BMD in the forearm was not examined. These are the limitations of this study. 

In conclusion, parathyroidectomy provided more BMD gain in the lumbar spine and femoral neck in symptomatic PHPT compared to patients with asymptomatic PHPT 1 year after parathyroidectomy. Preparathyroidectomy iPTH was an independent predictor of BMD gain. Parathyroidectomy has a more favorable effect on bone in patients with symptomatic PHPT compared to patients with asymptomatic PHPT. Long-term prospective studies are required to investigate the effect of parathyroidectomy on bone in patients with PHPT.
